# Frequent Hand Washing for COVID-19 Prevention Can Cause Hand Dermatitis: Management Tips

**DOI:** 10.7759/cureus.7506

**Published:** 2020-04-02

**Authors:** Cristina Beiu, Mara Mihai, Liliana Popa, Luiza Cima, Marius N Popescu

**Affiliations:** 1 Oncologic Dermatology, Elias Emergency University Hospital, "Carol Davila" University of Medicine and Pharmacy, Bucharest, ROU; 2 Pharmacy, "Carol Davila" University of Medicine and Pharmacy, Bucharest, ROU; 3 Physical Medicine and Rehabilitation, "Carol Davila" University of Medicine and Pharmacy, Bucharest, ROU

**Keywords:** covid-19, prevention, hand dermatitis, contact dermatitis

## Abstract

Coronavirus disease 2019 (COVID-19) continues to spread globally, outpacing the capacity and resources of health systems worldwide. A therapeutic vaccine is not yet on the rise, and preventive measures are the current approach to restraint the transmission of cases. As the virus is highly contagious via respiratory route (droplets from infected persons, widely spread by coughing or sneezing) and via contact with contaminated surfaces, community transmission and spread can be decreased through the practice of regular and diligent hand hygiene. Frequent hand washing implies a prolonged exposure to water and other chemical or physical agents and may induce several pathophysiologic changes, such as epidermal barrier disruption, impairment of keratinocytes, the subsequent release of proinflammatory cytokines, activation of the skin immune system, and delayed-type hypersensitivity reactions. Adverse dermatologic effects, such as excessive skin dryness or even contact dermatitis (particularly the irritant subtype and, to a lesser extent, the allergic subtype), can occur, especially in individuals with a history of atopic dermatitis. These skin conditions are perfectly manageable, and applying a moisturizer immediately after washing hands or after using a portable hand sanitizer is the cornerstone in preventing the development of eczematous changes in the hands. In the current global context, the potential occurrence of these dermatological adverse events should in no way cause people to deviate from strict hand hygiene rules.

## Introduction and background

COVID-19 stands for “coronavirus disease 2019,” and it refers to an outbreak of acute respiratory infection caused by a novel coronavirus. The specific coronavirus strain was initially referred to as 2019-nCoV (2019 novel coronavirus) and finally designated as SARS-CoV-2 (severe acute respiratory syndrome coronavirus 2). It was first identified in late 2019 in the city of Wuhan, Hubei Province of China, and it rapidly spread throughout other Eastern countries (e.g. South Korea, Japan, Iran) as well as Europe and the United States [1]. On March 2020, the WHO (World Health Organization) declared the COVID-19 outbreak a global pandemic and all countries were urged to undertake effective measures for reducing transmission [2].

Vaccines active against COVID-19 are not available, and ongoing prospects in formulating and developing preventive or therapeutic vaccines against SARS-CoV-2 are limited [3]. In this context, public health actions to prevent transmission are crucial in slowing the spread of the pandemic. One of the essential recommendations that the WHO has issued for the populous is to wash their hands frequently and correctly. In the process, intensified hand washing may generate various changes in skin texture and even hand dermatitis.

This article aims to review the potential dermatological adverse effects that may arise due to frequent hand washing, as well as practical tips for preventing these uncomfortable skin reactions. All clinical images included in the review section of the article were taken in the Department of Oncologic Dermatology of Emergency University Hospital “Elias” in Bucharest, using a digital camera (Nikon D3300; Nikon Corporation, Tokyo, Japan). 

## Review

**From frequent hand washing to hand dermatitis**

Frequent hygienization of hands may generate various changes in skin texture, ranging from the development of cutaneous xerosis (dryness of the skin) up to irritant contact dermatitis (ICD) or, rarely, even allergic contact dermatitis (ACD). Overall, these skin disorders are induced by various physical, chemical, and immunological mechanisms. When measures of diligent hand hygiene are implemented, these mechanisms may be activated mainly by the following circumstances.

**Figure 1:**
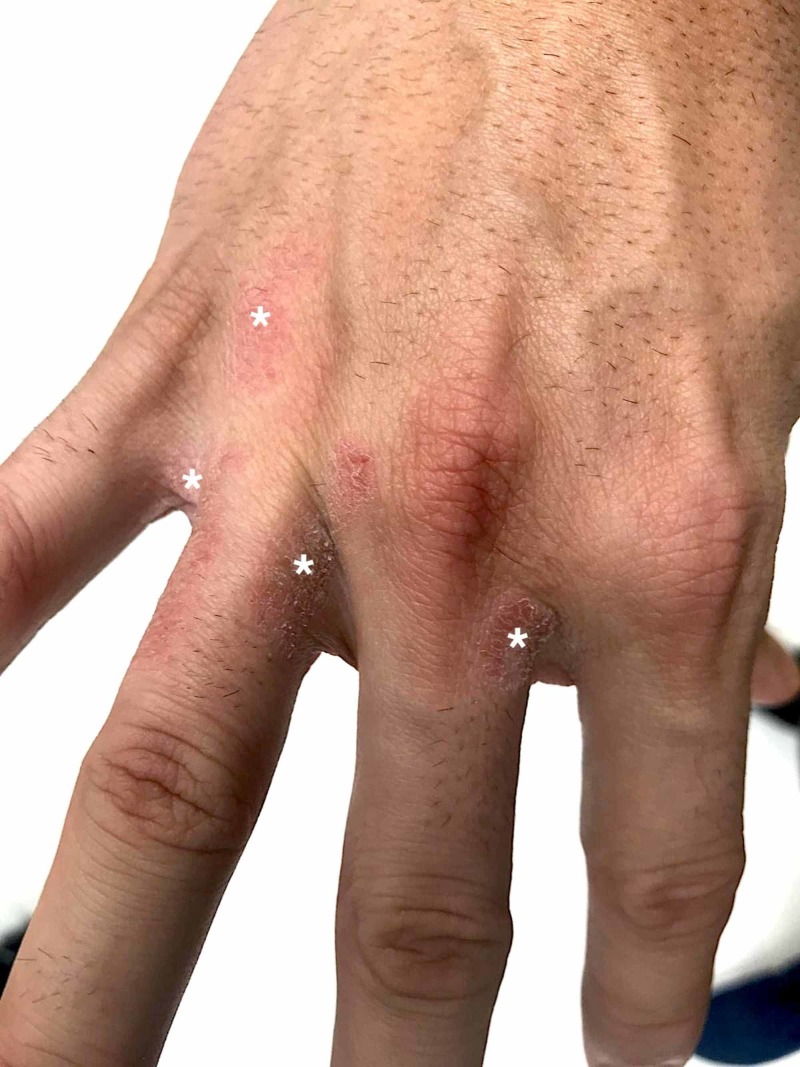
Clinical characteristics of irritant contact dermatitis in a 38-year-old patient who adopted frequent hand washing habits a month ago, as a preventive measure for COVID-19 spread, without using moisturizers. The white asterisks (*) highlight several ill-defined, xerotic (dry), erythematous scaly patches on the dorsum of the hands, fingertips, and finger webs, which progress to lichenification (skin thickening).

**Figure 2:**
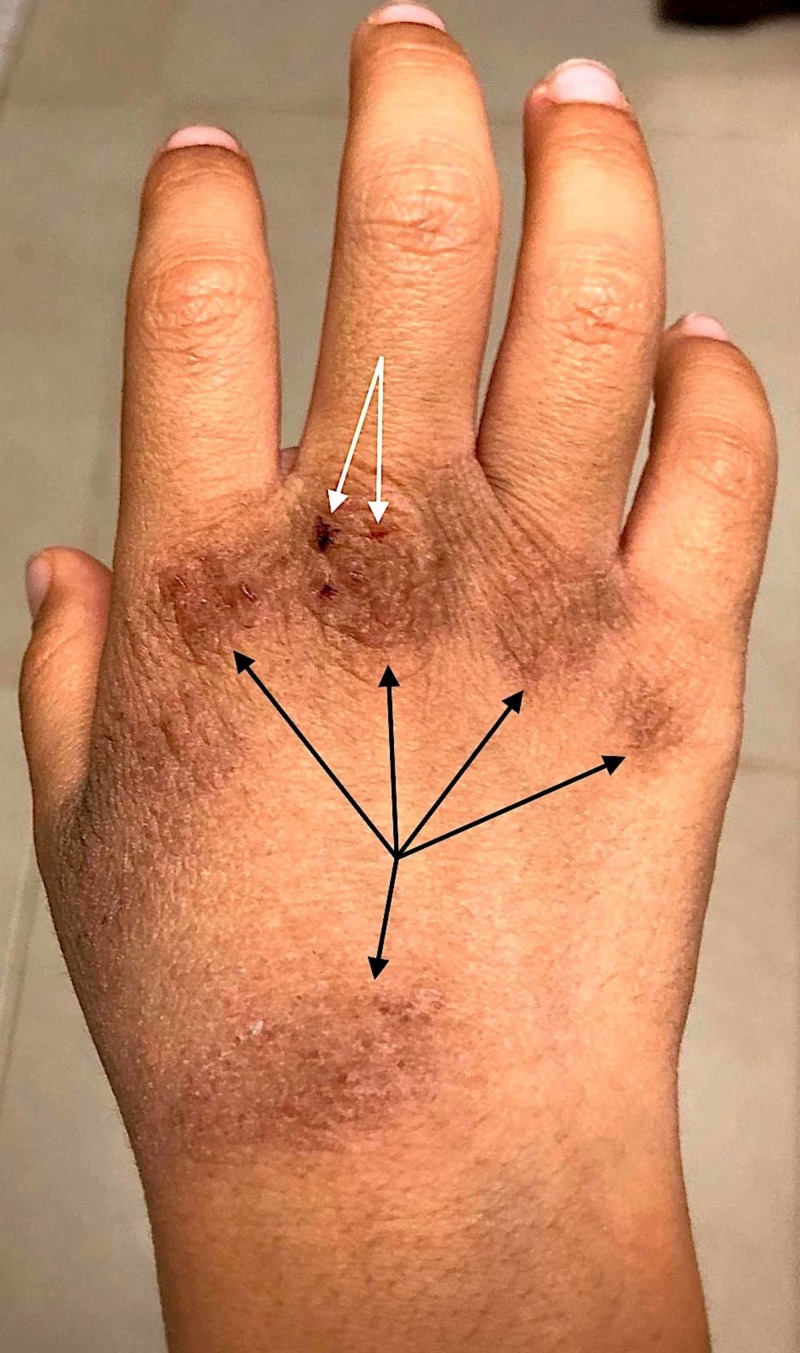
Eczematous skin changes on the hand of a 19-year-old Asian male, with a personal history of atopic dermatitis. The patient initiated preventive hand washing measures two months ago, without hydrating the hands afterward and developed severe skin dryness, fissuring (white arrow), and scaling.  Also the irritant-induced changes have progressed to hyperkeratosis and acanthosis (black arrows), highlighting the cumulative exposure.

**Figure 3:**
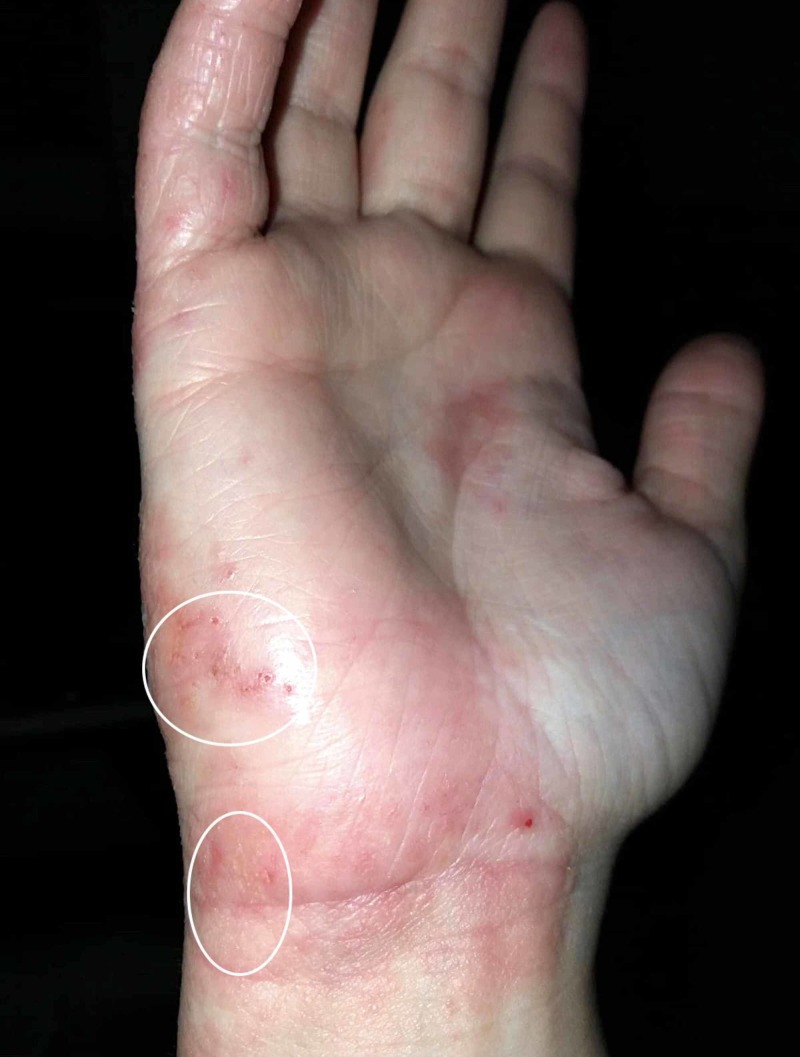
Clinical features of allergic contact dermatitis. Confluent, erythematous, scaly itchy patches, with small vesicles (highlighted in the white circles), on the hands of a patient with allergic reaction induced by chemicals in detergents and soaps.

1. Prolonged skin exposure to water and humid environment: It creates extensive swelling of stratum corneum (the skin's outermost layer) and disruption in the ultrastructure of intercellular lipids, and heightens the skin’s permeability and sensitivity to physical or chemical irritants [4]. In addition, prolonged wearing of protective gloves can generate excessive sweating and increased humidity, thus further increasing the inflammatory response elicited towards irritants.

2. Repeated use of soaps, surfactants, detergents, or solvents: These substances used for domestic cleaning are weak irritants and are usually very well tolerated. Nevertheless, repeated exposure to these substances can lead to chronic cumulative ICD (Figure 1), mainly due to their capacity to remove skin surface lipids, damage skin proteins, denature epidermal keratin, and even induce alteration of the cell membrane of keratinocytes [5]. Furthermore, patients with a personal or family history of atopic dermatitis have a chronically dysfunctional cutaneous barrier that increases their sensitivity to skin irritants (Figure 2) [6]. Rarely, some individuals may even develop ACD (Figure 3), a T-cell-mediated, delayed-type hypersensitivity reaction, to an ingredient in a hand hygiene-related product, such as soaps or detergents [7].

3. Repeated use of alcohol-based hand sanitizer: The WHO states that using hand rubs that contain at least 60% alcohol is a reasonable alternative if water and soap are not available, and as long as the hands are not visibly dirty [8]. Frequent use of these products can also result in skin dryness and irritation. On the other hand, allergy against alcohol itself is unknown and ACD attributable to other compounds added to alcoholic hand gels is extremely rare. This was illustrated by the experience of a large hospital in Switzerland where health workers routinely used a commercial alcohol-based hand sanitizer for 10 years, without reporting any allergic reactions to the product [9]. 

Management tips

In these times, it is very important to adapt our hand washing habits to ensure efficient protection against the spread of COVID-19 while lowering the risk of skin adverse reactions. In this respect, we find very useful the adoption of the following protective measures.

1. As the WHO recommends, hands should be washed thoroughly (including fingernails, interdigital web spaces, wrists) for at least 20 seconds, using lukewarm water and soap, particularly after being in public areas, before meals, after coughing or sneezing, after using the toilet, and whenever the hands are dirty.

2. After washing, we advise rinsing the hands by using gentle maneuvers, without causing a physical irritation to the skin.

3. Applying moisturizing skin care products after hand cleansing is the essential step in keeping the skin hydrated and preventing further abnormal skin reactions. These hydrating products should be liberally applied, multiple times per day, particularly immediately after hand washing.

4. There are several subtypes of moisturizers but to efficiently improve the quality of the skin barrier it is largely indicated to combine humectants with occlusive emollients. Humectants (e.g., topical urea, propylene glycol) are capable of attracting water to the stratum corneum from the environment and from the deeper layers of the skin. Occlusive emollients (e.g., petrolatum-based products, lanolin, mineral and vegetable oils, waxes) prevent water loss and alleviate irritation. A combination of the two is useful for attracting and sealing water at the level of the corneum layer and soothing the skin.

5. Thick greasy creams and ointments (e.g., petroleum jelly) provide higher protection against xerosis than lotions. To reduce the risk of contact sensitization, it is highly recommended to use fragrance-free and hypoallergenic products.

6. When soap and water are not available, the CDC advises that the use of alcohol-based hand sanitizers (that contain at least 60% alcohol), is an effective alternative in destroying the virus. Since these can be irritating, it is important to hydrate the skin immediately after. Applying a moisturizing cream afterward does not interfere in any way with the properties and efficiency of this type of sanitizers.

7. For individuals working with protective gloves, it is highly recommended to wash hands and apply moisturizer whenever gloves are taken off. Also, to lower the humidity, they should be changed systematically and applied only on dry hands.

8. For people with highly sensitive skin, which easily develop disturbing forms of dermatitis, short courses of topical corticosteroids may be used to reduce the signs and symptoms of inflammation.

## Conclusions

Compliance with hand hygiene recommendations is essential in preventing the spread of COVID-19 and, under no circumstances, should be diminished by the eczematous changes that may occur in the hands. In this context, the potential development of hand dermatitis is preventable and manageable by using the appropriate skin care products. Regular skin hydration is a key component in preventing hand dermatitis as a consequence of frequent washing.
